# Interp-SUM: Unsupervised Video Summarization with Piecewise Linear Interpolation

**DOI:** 10.3390/s21134562

**Published:** 2021-07-02

**Authors:** Ui-Nyoung Yoon, Myung-Duk Hong, Geun-Sik Jo

**Affiliations:** Artificial Intelligence Laboratory, Department of Electrical and Computer Engineering, Inha University, Incheon 22212, Korea; entymos@hotmail.com (U.-N.Y.); hongmyungduk@gmail.com (M.-D.H.)

**Keywords:** video summarization, reinforcement learning, unsupervised learning, piecewise linear interpolation

## Abstract

This paper addresses the problem of unsupervised video summarization. Video summarization helps people browse large-scale videos easily with a summary from the selected frames of the video. In this paper, we propose an unsupervised video summarization method with piecewise linear interpolation (Interp-SUM). Our method aims to improve summarization performance and generate a natural sequence of keyframes with predicting importance scores of each frame utilizing the interpolation method. To train the video summarization network, we exploit a reinforcement learning-based framework with an explicit reward function. We employ the objective function of the exploring under-appreciated reward method for training efficiently. In addition, we present a modified reconstruction loss to promote the representativeness of the summary. We evaluate the proposed method on two datasets, SumMe and TVSum. The experimental result showed that Interp-SUM generates the most natural sequence of summary frames than any other the state-of-the-art methods. In addition, Interp-SUM still showed comparable performance with the state-of-art research on unsupervised video summarization methods, which is shown and analyzed in the experiments of this paper.

## 1. Introduction

With the exponential increase in online video, video summarization research has become attractive because it facilitates large-scale video browsing and efficient video analysis [1]. Many research results have been presented with various methods to summarize video to the key shots or keyframes with these contents: frequently occurring content, newly occurring content, or interesting and visually informative content [2,3,4].

Recently, the reinforcement learning (RL) based unsupervised video summarization method outperformed in results with an explicit reward function to select keyframes [4]. However, it is still difficult to make a natural video summary, by training the summarization network using RL, because of the high dimensional action space. In the case of high dimensional action space, the number of actions for selecting a keyframe from the video is very large, so it is difficult to select an action that guarantees high reward from the numerous actions due to the computational complexity. For this reason, the high dimensional action space causes high variance problems for training the network with RL [5,6].

**Figure 1:**
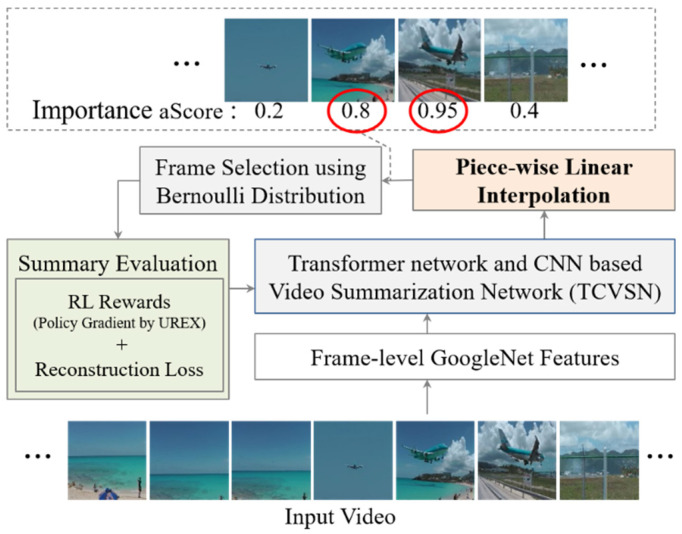
Overview: Our goal is to select keyframes with high importance scores to summarize the video based on the reinforcement learning method.

In this paper, we propose an unsupervised video summarization method with piecewise linear interpolation (Interp-SUM). Interp-SUM is a method to predict importance scores by interpolating the output of a deep neural network. The piecewise linear interpolation method is utilized to mitigate the high variance problem and to generate a natural sequence of summary frames. Figure 1 shows an overview of our method to summarize video to predict the importance score of the frames and select keyframes as a summary of the video using the score.

We extract visual features from the frame images of the video using GoogleNet [7]. GoogleNet is a deep convolutional neural network, also known as Inception. The GoogleNet has the Inception modules that perform different sizes of filters and concatenate them for the next layer. To generate the video summary well, we present the Transformer network and CNN based video summarization network (TCVSN), which is motivated by Conformer Network (Convolution-augmented Transformer) [8]. TCVSN is composed of a transformer encoder module, convolution module, and feed-forward module. Our network learns the global and local context of the video with the modules and summarizes the video. The last several sequences of the output of the network are calculated to the importance score candidate by using the fully connected layer and the sigmoid function. The candidate is interpolated to the importance score of the frames.

Especially in the case of the high dimensional action space, the reward is changing while training because there are many cases where different actions are selected. Therefore, the variance of the gradient estimate is calculated with the log probability of the action, and the reward is increased. When the frames are selected, using the interpolated importance score, nearby frames with similar scores are selected together in high probability. In other words, the number of variants of the action is decreased, and it makes an effect of reducing the action space. A natural sequence of summary frames is generated by selecting adjacent frames with high importance scores together, as shown in experiments. The high variance problem is mitigated by the reduced action space.

After interpolation, we convert the importance score to 0 or 1 frame-selection action using Bernoulli distribution. Using the action, we select keyframes as a summary, then evaluate how diverse and representative the summary is by using reward functions. Then, we compute the objective function proposed in exploring the under-appreciated reward (UREX) method with the reward and log probabilities of the action [9]. UREX objective function is a sum of expected reward and RAML objective function, which learns a policy by optimizing the reward. Finally, we calculate modified reconstruction loss to promote the representativeness of the summary.

The main contributions of this paper are as follows:

1. We propose an unsupervised video summarization method, with piecewise linear interpolation, to mitigate the high variance problem and to generate a natural sequence of summary frames. It reduces the size of the output layers of the network. It also makes the network learn faster because the network needs to predict only the short importance score in this method.
2. We present Transformer network and CNN based video summarization network (TCVSN) to generate importance score well.
3. We develop a novelty reward that measures the novelty of the selected keyframes.
4. We develop the modified reconstruction loss with random masking to promote the representativeness of the summary.

## 2. Background and Related Work

### 2.1. Video Summarization

Video summarization methods are divided into the supervised learning-based method and the unsupervised learning-based method [2,3,4,10,11,12]. The supervised learning-based method uses the training dataset to train the model to find the best predictions of the model that minimize the loss calculated with the ground truth. The ground truth datasets include annotations which are importance scores for every frame or every shot of the video [13,14].

In [10], they presented an LSTM-based model with a determinantal point process (DPP) that encodes the probability to sample dataset for learning representativeness and diversity. In [11], DTR-GAN is proposed, which uses Dilated Temporal Relational (DTR) units for capturing temporal relations among video frames and LSTM to predict the frame-level importance scores, after which the discriminator generates representations of summaries for training. In [3], attention mechanism is used for training the importance score by using the encoder-decoder network. This score is calculated with encoder’s out and decoder’s last hidden state using their scoring function.

However, the problem with the supervised learning-based method is that it is very difficult to create a large-scale video summarization dataset, including videos of various domains and situations, with human annotations [2]. On the other hand, the unsupervised learning-based method trains the model without the ground truth dataset. There are many researchers who use this method by casting the video summarization problem as a keyframe selection problem [2,4,15].

In [2], VAE and GAN based models are proposed. The selector LSTM selects a subset of frames from the input feature, then inputs to Variational Autoencoder (VAE) for reconstructing the summary video. Discriminator distinguishes between the reconstructed video and the original video. For training the model, four different loss functions are used. In [15], VAE and GAN are used with architecture similar to that in [2]. However, the model adopts a cycle generative adversarial network for preserving the information of the original video in the summary video. In [16], they proposed an unsupervised SUM-FCN. FCN is popular semantic segmentation architecture with temporal convolution converted from spatial convolution. They select frames using the output score of the decoder and calculate the loss with a repelling regularizer for learning diversity.

### 2.2. Policy Gradient Method

The policy gradient method is a popular and powerful policy-based reinforcement learning method [17,18,19,20]. It optimizes the parameterized policy using the reward (cumulative reward in an episode) by a gradient descent method, such as SGD [21]. The policy gradient method, especially, operates by increasing the maximized log probabilities of the actions and reward from the action generated by the policy [5]. However, the policy gradient method has several problems, such as the low sample efficiency problem [22]. It means that the agent requires many more samples (experience), for learning actions in the environment (states), than humans. Another problem is the high variance of the estimated gradient. This problem is caused by the high dimensional action space and long-horizon problem [23], which means a hugely delayed reward for a long sequence of decisions to find a goal. In our proposed method, we present the piecewise linear interpolation-based video summarization method to reduce variance with the effect of reducing action space.

## 3. Unsupervised Video Summarization with Piecewise Linear Interpolation

**Figure 2:**
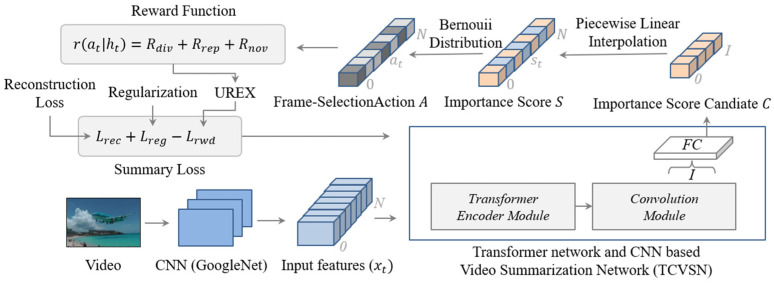
Interpolation-based video summarization framework.

We formulate video summarization as a frame selection problem with importance scores trained by the video summarization network. In particular, we develop a video summarization network, which is a parameterized policy to predict the importance score candidates to be interpolated to the importance scores. In other words, the importance score is frame-selection probability. The scores converted as frame-selection actions to select keyframes by using Bernoulli distribution, as shown in Figure 2.

### 3.1. Generating Importance Score Candidate

We first extract visual features ${\left\{x_{t}\right\}}_{t=1}^{N}$ from the frame images in the given video using GoogleNet [6], which is a deep convolutional neural network trained with an ImageNet dataset. Feature extraction is needed to capture the visual description of the frame images and visual differences among them [24]. To train the video summarization network, as shown in Figure 2, we input the sequence of frame-level features.

**Figure 3:**
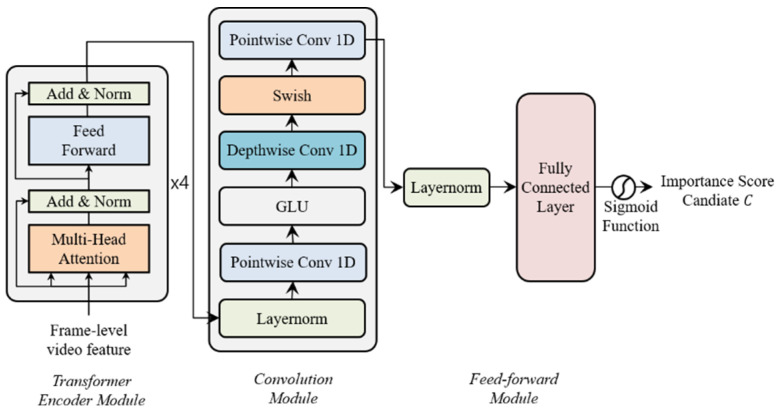
Transformer network and CNN based Video Summarization Network (TCVSN).

We propose Transformer network and CNN based video summarization network (TCVSN) to increase video summarization performance as shown in Figure 3 which is composed of a transformer encoder module, convolution module, and feed-forward module. Our network is motivated by the Conformer network [7]. The Conformer network was proposed for audio speech recognition (ASR). The main idea of the Conformer network is that the transformer network captures the global context of the audio sequence and the convolutional neural network (CNN) captures local context. We follow this idea in this paper, but we changed and simplify network architecture. We use the original transformer encoder network, proposed in [8], to replace the multi-head self-attention module of the Conformer network. We adapt the convolution module of the Conformer network without batch normalization and dropout. We propose our network with shallow architecture without residual connections. Because we train the network with smaller datasets than the audio datasets. In the feed-forward module, we use layer normalization (LayerNorm) [25] and a fully connected network with the sigmoid function to calculate the importance score candidate C. Especially in our network, key properties (re-scaling, re-centering) of layer normalization are very important to distribute the importance score candidate values evenly. Because without layer normalization, the importance score candidate values tend to be concentrated in the middle (0.5) after the sigmoid function. Therefore, we can learn the representation of the video and visual differences among the frames in the video with our proposed network, which has hidden states ${\left\{h_{t}\right\}}_{t=1}^{N}$. The output of the network is the importance score candidate $C={\left\{c_{t}\right\}}_{t=1}^{I}$ to be interpolated to importance score $S={\left\{s_{t}\right\}}_{t=1}^{N}$ which is the frame-selection probability to select frames as a video summary. We choose the last time step sequence within I length of the output. Then, by using the fully connected (FC) layer and sigmoid function, we change the output, which is multi-dimension features to the 0 to 1 probabilities of the importance score candidate.

### 3.2. Importance Score Interpolation

**Figure 4:**
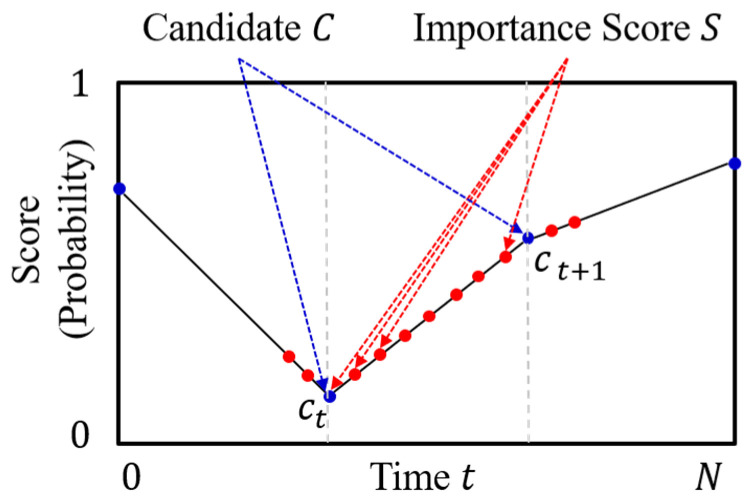
Piecewise linear interpolation method to interpolate the importance score candidate to the importance score.

Interpolation is a type of estimation method that guesses the new data points within the range of the known discrete data point. We first align the importance score candidate to fit the input size N of the frame sequence at equal intervals. We interpolate the candidate (C) to the importance score (S) using piecewise linear interpolation, as shown in Figure 4. Piecewise linear interpolation connects the importance score candidate with the linear line and calculates intermediate values on the line [26]. After interpolation, we find the importance scores of each frame of the video, which is the frame-selection probabilities.

The interpolation method is proposed to reduce the computational complexity of the video summarization network and to make the network learn faster. Because the network needs to predict only I length of the importance score candidate, not all sequences [27]. The interpolation method mitigates the high variance problem and facilitates to generate a natural sequence of summary frames. Especially, in the case of the high dimensional action space, the reward of the action is changing in every step. Because we use Bernoulli distribution for selecting frames. Therefore the variance of the gradient estimate which is calculated with the reward is increased. However, when the frames are selected using the interpolated importance score, near frames with similar scores are selected as shown in Figure 4. It makes an effect of reducing the action space and mitigates the high variance problem.

### 3.3. Training with Policy Gradient

After interpolation, we convert the importance score S, that is frame-selection probabilities to frame-selection action $A=\{a_{t}|a_{t}\in \left\{0,\;1\right\},\;t=1,\ldots ,N\}$ using the Bernoulli distribution. If the frame-selection action of a frame is equal to 1, we select this frame as the keyframe as a summary of the video.

(1) $$A\;\sim \;Bernoulli\left(a_{t};s_{t}\right)=\{\begin{array}{cc} s_{t}, & for\;a_{t}=1\\ 1-s_{t}, & for\;a_{t}=0 \end{array}$$

We sample the frame-selection action from the importance score using Bernoulli distribution to evaluate the policy efficiently. By using the reward function, we evaluate the quality of the variants of the summary generated by frame-selection action for several episodes, and we find log probabilities of the action and reward at the end of the episode. In this paper, we use the diversity and representativeness reward function, which are proposed in [4]. We expect that sum of the diversity reward ($R_{div}$) and the representativeness reward ($R_{rep}$) are maximizing while training.

The diversity reward ($R_{div}$) (2) measures the dissimilarity among the selected keyframes using frame-level features. Based on reward, the policy is trained to generate the frame-select action for selecting diverse frames as keyframes. To prevent the problem that the reward function calculates dissimilarity of frames far from each other, we limit the temporal distance to 20 for the calculation because they are needed to keep the storyline of the video. This reduces the computational complexity.

Let the indices of the selected frames be $ℐ=\{{𝒾}_{k}|a_{{𝒾}_{k}}=1,\;k=1,2,\ldots ,\;\left|ℐ\right|\}$, then the diversity reward is:

(2) $$R_{div}=\frac{1}{\;ℐ\left(\;ℐ-1\right)}\underset{t\in \;ℐ}{\sum }\underset{\begin{array}{c} t^{\prime }\in ℐ\\ t\neq t^{\prime } \end{array}}{\sum }\left(1-\frac{x_{t}^{T}x_{t^{\prime }}}{||x_{t}||{}_{2}||x_{t^{\prime }}||{}_{2}}\right)$$

The representativeness reward ($R_{rep}$) (3) measures the similarity of the selected frame-level features, and all frame-level features of the original video, and generates a summary of the video that represents the original video.

(3) $$R_{rep}=exp\left(-\frac{1}{N}\overset{N}{\underset{t=1}{\sum }}\underset{t^{\prime }\in ℐ}{\min}||x_{t}-x_{t^{\prime }}||{}_{2}\right)\;$$

The novelty reward ($R_{nov}$) (4) measures the novelty of the selected keyframes. In other words, this reward function measures the dissimilarity between the selected keyframe and the previous several keyframes. This reward function calculates the L2 Loss between the features of the randomly picked keyframe and the features of the previous several keyframes. Let w be the window size and r be the list of randomly picked 10% keyframes from the actions, (4) is the novelty reward function.

(4) $$R_{nov}=L2\;Loss\;\left(x_{r}-\frac{1}{w}\overset{r-1}{\underset{t=r-w}{\sum }}x_{t}\right)\;$$

To train the parameterized policy $\pi_{\theta }$, which is the video summarization network, we use the policy gradient method with the exploration strategy of exploring under-appreciated reward (UREX) method [9]. If the log probability of the action, under current policy, underestimates its reward, then action will be explored more by the proposed exploration strategy.

To compute the objective function (5), we first calculate the log probability $\log\;\pi_{\theta }(a_{t}\;|\;h_{t})$ of the action $\;\pi_{\theta }(a_{t}\;|\;h_{t})$ and the reward $r(a_{t}\;|\;h_{t})=R_{div}+R_{rep}+R_{nov}$ in J episodes. At the end of the episode, we keep the log probability of the action and the reward for computing the objective function. We approximate the expectation by calculating rewards from variants of frame-selection action for several episodes on the same video [4]. Expectation, calculated over all variants of frame-selection action, is very difficult within a short time, moreover, longer frame sequence input makes it more difficult to calculate.

$O_{UREX}$ is an objective function for training the network. The objective function is the sum of the expected reward and $O_{RAML}$. $O_{RAML}$ is reward augmented maximum likelihood (RAML) objective function to optimize reward proposed in [28]. At the beginning of training, the variance of importance weights is too large, so they are combined with the expected reward. To approximate $O_{RAML}$, we sample jth actions and compute the set of normalized importance weights using softmax, $softmax(r(a_{t}^{\left(j\right)}\;|h_{t})\;)/\tau -\log\pi_{\theta }(a_{t}^{\left(j\right)}|\;h_{t})$. τ is a regularization coefficient to avoid excessive random exploration.

(5) $$O_{UREX}\left(\theta ;\tau \right)=E_{h\sim p\left(h_{t}\right)}\left\{\underset{a\in A}{\sum }R(a_{t}\;|\;h_{t})\right\}\;$$

(6) $$R(a_{t}\;|\;h_{t})=\pi_{\theta }\left(a_{t}\;|\;h_{t}\right)\;r\left(a_{t}\;|\;h_{t}\right)+O_{RAML}\;$$

(7) $$O_{RAML}=\tau \pi_{\tau }^{*}\left(a_{t}\;|\;h_{t}\right)\;\log\pi_{\theta }\left(a_{t}\;|\;h_{t}\right)$$

We use the baseline, which is an important method for policy gradient, to reduce variance and to improve computational efficiency. The baseline is calculated as the moving average of rewards experienced so far and updated at the end of the episode. Our baseline ℬ is the moving average reward of each video ($v_{i}$).

(8) $$ℬ=1/J\times \underset{j\in J}{\sum }r\left[v_{i}\right]$$

(9) $$L_{rwd}=O_{UREX}\left(\theta ;\tau \right)-ℬ$$

Based on the policy gradient, we maximize $L_{rwd}$ to train the parameterized policy, video summarization network.

### 3.4. Regularization and Reconstruction Loss

We use the regularization term $L_{reg}$ which is proposed in [4] to control frame-selection action. If more frames are selected as keyframes for video summarization, the reward will increase with our reward function. Without a regularization term, the interpolation score, which is frame-selection probability, can be increased to 1 or decreased to 0 while training for maximizing the reward. We use a value (0.01) to avoid overfitting and the percentage of frames to be selected value (0.5).

(10) $$L_{reg}=0.01\times {\left(\frac{1}{N}\times \overset{N}{\underset{1}{\sum }}s_{t}-0.5\right)}^{2}\;$$

We introduce the modified reconstruction loss with random masking to promote the representativeness of the summary for training our video summarization network. Using the importance score S, we multiply the score $s_{t}$ by input frame-level features $x_{t}$ to calculate the representativeness of the frame-level features at time t. If a score is high at time t, the frame-level features at time t may represent the video attention. We mask 20% of the input features $x_{t}^{M}$ with zero, randomly, to prevent the problem with the importance score $s_{t}$ close to 1. D is the dimension size of the input feature (1024) to resize the value of the $L_{rec}$ because the sum of the squared difference of $x_{t}^{M}$ and $x_{t}\times s_{t}$ is too big to use.

(11) $$L_{rec}=\frac{1}{D}\times \sum {\left(x_{t}^{M}-x_{t}\times s_{t}\right)}^{2}$$

After we compute all of the loss function, we finally calculate the loss $L_{summary}$ and do the backpropagation.

(12) $$L_{summary}=L_{reg}+L_{rec}-L_{rwd}$$

Algorithm 1 is about the training procedure of the unsupervised video summarization method with the interpolation method.
**Algorithm 1.** Training Video Summarization Network.1: Input: Frame-level features of the video

2: Output: TCVSN parameters (θ)

3:

4: for number of iterations do

5:  $x_{t}$ ← Frame-level features of the video

6:  C ← TCVSN($x_{t}$)% Generate candidate

7:  S ← Piecewise linear interpolation of C

8:  A ← Bernoulli(S)% Action A from the score S

9:  % Calculate Rewards and Loss using A and S

10:   % Update using policy gradient method:

11:   {θ} $\overset{+}{\leftarrow }$ $-\nabla \left(L_{reg}+l_{rec}-L_{rwd}\right)$% Minimization

12: end for

### 3.5. Generating Video Summary

To test TCVSN, our video summarization network, we calculate the shot-level importance scores by averaging the frame-level importance scores within the shot. To generate key shots for the dataset, many video summarization researches [2,4,15] use Kernel Temporal Segmentation (KTS), which detects change points, such as shot boundaries. To generate the video summary, we select key shots over the top 15% of the video length sorted by the score. This step is necessary for the 0/1 Knapsack problem, as described in [4].

## 4. Experiments

### 4.1. Dataset

We evaluate our method on two datasets: SumMe [13] and TVSum [14]. SumMe consists of 25 videos covering various topics such as airplane landing and extreme sports. Each video is about 1 to 6.5 min long and shot with various types of camerawork. Frame-level importance scores for each video annotated by 15 to 18 human annotators. TVSum consists of 50 videos of various topics such as news, documentary, vlog. Each video has shot-level importance scores annotated by 20 human annotators. Each video in TVSum varies from 2 to 10 min.

### 4.2. Evaluation Setup

For a fair comparison with other methods, we follow the evaluation method, which is used in [4] to compute the F-measure as the performance metric. We first calculate the precision, the recall based on the result, and we compute the F-measure. Let G be the generated shot level summary by the proposed video summarization method and A be the user annotated summary in the dataset. The precision and recall are calculated based on the amount of temporal overlap between G and A as below.

(13) $$Precision=\frac{Duration\;of\;overlap\;between\;G\;and\;A}{Duration\;of\;G}$$

(14) $$Recall=\frac{Duration\;of\;overlap\;between\;G\;and\;A}{Duration\;of\;A}$$

(15) $$F-measure=\frac{2\times Precision\times Recall}{Precision+Recall}\times 100\%$$

For a fair comparison, we use 5-fold cross-validation to find the performance of the network. We test our network for five different random splits and take the result of the average performance. Specifically to create random splits, we split the videos in the dataset into a training dataset and a validation dataset. We calculated the F-measure using the validation dataset.

### 4.3. Implementation Details

We develop the video summarization method using the Pytorch 1.7.0 version. Proposed our network has three modules. First is the Transformer encoder module, which is composed of a stack of 4 layers with 512 hidden units and 8 attention heads. The second is the convolution module, which contains 1d depthwise separable convolution with 32 kernel size and 1024 channel size. The third is the feed-forward module, which reduces dimension from 1024 to 1. We use the Adam optimizer [21] to train the network [8] with a learning rate of 0.00001. We train the network for 200 epochs and pick the best model at 115 epochs.

### 4.4. Quantitative Evaluation

We first compare different variants of our method. Then, we compare our method with several unsupervised video summarization methods.

**Figure 5:**
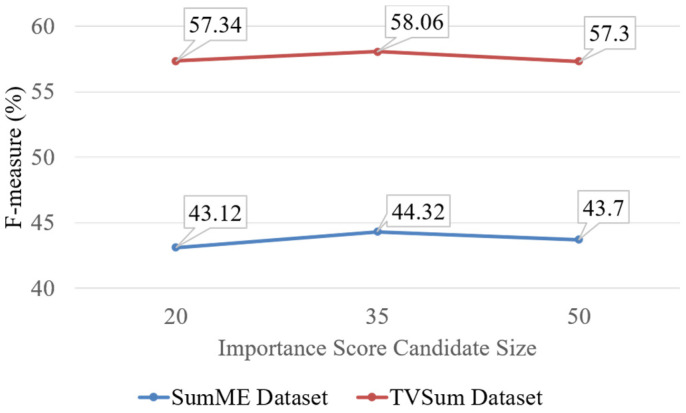
Average result (%) for different importance score candidate sizes (20, 35, 50) to be interpolated to importance score on SumMe and TVSum dataset.

As shown in Figure 5, we evaluate the performances of our methods with importance score candidate size on SumMe and TVSum datasets. Our method shows the best performance on both datasets when we set the importance score candidate size to 35. In our analysis, if the importance score candidate size is 50, performance is high when video duration is long, such as video #10 and video #15 on SumMe dataset. If the importance score candidate size is 35, performance is best when the video duration is average in the SumMe dataset. However, if video duration is short, performance is very poor and even the importance score candidate size is large or short. Therefore, based on the analysis, we need to develop a new method that can find the best importance score size automatically to improve performance.

Table 1 shows the performance of different variants of the proposed method. We first compare the proposed method with or without interpolation. In the case of our method without interpolation, performance is lower than the proposed method with interpolation on both datasets. We think the lower performance is due to the high variance problem that slows learning speed because there are too many summaries to generate from an action. In the case of our method without UREX, the result indicates that performance improvement with UREX is smaller than the other proposed methods. Nonetheless, we think that UREX is important for our method to get the best performance with an efficient exploration strategy. In the case of our method without reconstruction loss, the result indicates that the proposed reconstruction loss highly improves summarization performance. We think that the reconstruction loss helps to learn the network efficiently when reinforcement learning is less efficient because of the high variance problem.

Table 2 shows the comparison between our proposed method and state-of-the-art unsupervised video summarization methods. Comparing with other unsupervised methods, our proposed method shows almost better performance on all datasets. Specifically, the summarization performance of our method outperforms the DR-DSN, which is also based on reinforcement learning and uses the same representativeness and diversity reward function [4]. However, Tessellation and SUM-GAN-AAE showed better performance on each dataset. SUM-GAN-AAE especially showed similar performance with our proposed method. Nevertheless, our proposed method is novel in a hugely different way from SUM-GAN-AAE. SUM-GAN-AAE uses an adversarial autoencoder with the attention-based encoder-decoder network.

### 4.5. Qualitative Evaluation

We analyze the effects of different components of our method with the following variants:

1. Interp-SUM is our proposed method with no changes.
2. Interp-SUM w/o UREX which does not use the exploring under-appreciated reward (UREX) method. This variant uses the method proposed in [4].
3. Interp-SUM w/o Recon. Loss which does not use the modified reconstruction loss that we presented.
4. Interp-SUM w/o Interp. which does not use the piecewise linear interpolation method. Our network is trained to predict the importance score directly.

**Figure 6:**
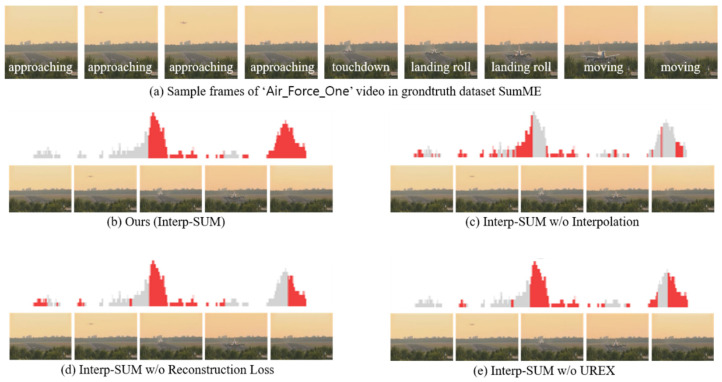
Visualized importance scores and sampled summary frame images of the video which is ‘Air Force One’ in the SumMe dataset [13]. The gray bars are the groundtruth summary importance scores. Red bars are the top 1/3 of importance scores from the generated importance scores by different variants of our approach.

We visualize the qualitative results of different variants of our method with example images. Figure 6 presents sample frames with summary scores for different variants of our method. The gray bars are the ground truth summary importance scores. Red bars are the top 1/3 of generated importance scores by different variants of our approach. As presented in Figure 6b, Interp-SUM enables selecting adjacent frames of a keyframe predicted by a high score. Because scores of the neighboring frames are similar. This is an advantage of the interpolation method to retain relations between neighboring frames while training for selecting keyframes. Based on the interpolation method, the network generates a more natural sequence of summary frames than the network without the interpolation method, and the keyframes in the main content are selected. As presented in Figure 6c, when we do not use a piecewise linear interpolation method, it is even difficult to select the surrounding frames of the keyframe that has high importance score. Because the importance scores of the near frames directly generated from the network are not as similar as interpolated importance scores of the near frames. It means that the near frames are not selected often without interpolation. In the case of Interp-SUM without reconstruction loss, as shown in Figure 6d, we think that, since only reward function for learning representativeness of the video is not enough to train network, the frames with higher importance scores are less selected such as landing roll or moving. Interp-SUM without UREX, as shown in Figure 6e, shows almost the same performance as Interp-SUM, but as you can see, the last few important frames (moving) are not selected. We think that the result of Interp-SUM without UREX indicates that the UREX algorithm is also important to improve video summarization performance.

## 5. Conclusions

We proposed an unsupervised video summarization method with a piecewise linear interpolation method (Interp-SUM). We present the Transformer network and CNN-based video summarization network (TCVSN). We use the policy gradient method with UREX objective function in reinforcement learning for training the network. We introduced the interpolation method to mitigate the high variance problem and to generate a natural sequence of summary frames. In the experimental results, the interpolation method helped to generate a more natural sequence of summary frames and improves performance by mitigating high variance problems. We showed the best performance on medium-length videos when we choose interpolation size to 35. Specifically, the F-measure on SumMe is 47.68, and the F-measure on TVSum is 59.14. This result indicates that our proposed method showed comparable performance on both datasets with the compared methods. In the future, we plan to make a network to find the best interpolation size automatically. To mitigate the high variance problem more, we will use new interpolation methods and employ the state-of-the-art techniques of reinforcement learning.

## Figures and Tables

**Table 1:** Result (%) of variants of our proposed method tested on SumMe and TVSum.

Method	SumMe	TVSum
w/o Interpolation	42.32	55.32
w/o UREX	42.46	57.42
w/o Reconstruction loss	41.52	49.86
Ours (Interp-SUM)	**44.32**	**58.06**

**Table 2:** Result (%) of the comparison of unsupervised based methods tested on SumMe and TVSum. Our proposed method shows state-of-the-art performance.

Method	SumMe	TVSum
SUM-GANdpp [2]	39.1 (−)	51.7 (−)
DR-DSN [4]	41.4 (−)	57.6 (−)
SUM-FCNunsup [15]	41.5 (−)	52.7 (−)
Cycle-SUM [16]	41.9 (−)	57.6 (−)
Tessellation [29]	41.4 (−)	**64.1 (+)**
UnpairedVSN [30]	47.5 (−)	55.6 (−)
SUM-GAN-sl [31]	47.3 (−)	58.0 (−)
SUM-GAN-AAE [32]	**48.9 (+)**	58.3 (−)
Ours (Interp-SUM)	**47.68**	**59.14**

## Data Availability

Not applicable.

## References

[1] Ejaz N., Mehmood I., Baik S.W. (2013). Efficient visual attention based framework for extracting key frames from videos. J. Image Commun..

[2] Mahasseni B., Lam M., Todorovic S. Unsupervised Video Summarization with Adversarial LSTM Networks. Proceedings of the IEEE Conference on Computer Vision and Pattern Recognition.

[3] Ji J., Xiong K., Pang Y., Li X. (2019). Video Summarization with Attention-Based Encoder-Decoder Networks. IEEE Trans. Circuits Syst. Video Technol..

[4] Zhou K., Qiao Y., Xiang T. (2018). Deep Reinforcement Learning for Unsupervised Video Summarization with Diversity-Representativeness Reward. AAAI Conf. Artif. Intell..

[5] Wu C., Rajeswaran A., Duan Y., Kumar V., Bayen A.M., Kakade S., Mordatch I., Abbeel P. Variance Reduction for Policy Gradient with Action-Dependent Factorized Baselines. Proceedings of the International Conference on Learning Representations (ICLR).

[6] Szegedy C., Liu W., Jia Y., Sermanet P., Reed S., Anguelov D., Erhan D., Vanhoucke B., Rabinovich A. Going deeper with convolutions. Proceedings of the IEEE Conference on Computer Vision and Pattern Recognition (CVPR).

[7] Gulati A., Qin J., Chiu C.C., Parmar N., Zhang Y., Yu J., Han W., Wang S., Zhang Z., Wu Y. (2020). Conformer: Convolution-augmented Transformer for Speech Recognition. arXiv.

[8] Vaswani A., Shazeer N., Parmar N., Uszkoreit J., Jones L., Gomez A.N., Kaiser L., Polosukhin I. Attention is all you need. Proceedings of the Advances in Neural Information Processing Systems (NIPS).

[9] Nachum O., Norouzi M., Schuurmans D. (2016). Improving Policy Gradient by Exploring Under-Appreciated Rewards. arXiv.

[10] Zhang K., Chao W.L., Sha F., Grauman K. (2016). Video Summarization with Long Short-term Memory. Proceedings of the European Conference on Computer Vision (ECCV).

[11] Zhang Y., Kampffmeyer M., Zhao X., Tan M. DTR-GAN: Dilated Temporal Relational Adversarial Network for Video Summarization. Proceedings of the ACM Turing Celebration Conference (ACM TURC).

[12] Zhang K., Grauman K., Sha F. (2018). Retrospective Encoders for Video Summarization. Proceedings of the European Conference on Computer Vision (ECCV).

[13] Gygli M., Grabner H., Riemenschneider H., Gool L.V. (2015). Creating summaries from user videos. Proceedings of the European Conference on Computer Vision (ECCV).

[14] Song Y., Vallmitjana J., Stent A., Jaimes A. Tvsum: Summarizing web videos using titles. Proceedings of the IEEE Conference on Computer Vision and Pattern Recognition.

[15] Rochan M., Ye L., Wang Y. (2018). Video Summarization Using Fully Convolutional Sequence Networks. Proceedings of the European Conference on Computer Vision (ECCV).

[16] Yuan L., Tay F.E., Li P., Zhou L., Feng F. Cycle-SUM: Cycle-consistent Adversarial LSTM Networks for Unsupervised Video Summarization. Proceedings of the Thirty-Third AAAI Conference on Artificial Intelligence.

[17] Silver D., Lever G., Heess N., Degris T., Wierstra D., Riedmiller M. Deterministic Policy Gradient Algorithms. Proceedings of the 31st International Conference on International Conference on Machine Learning (ICML).

[18] Liu T. (2020). Compare and Select: Video Summarization with Multi-Agent Reinforcement Learning. arXiv.

[19] Song X., Chen K., Lei J., Sun L., Wang Z., Xie L., Song M. Category driven deep recurrent neural network for video summarization. Proceedings of the 2016 IEEE International Conference on Multimedia & Expo Workshops (ICMEW).

[20] Sutton R.S., McAllester D., Singh S., Masour Y. Policy gradient methods for reinforcement learning with function approximation. Proceedings of the 12th International Conference on Neural Information Processing Systems (NIPS).

[21] Kingma D.P., Ba J.L. Adam: A method for stochastic optimization. Proceedings of the 3rd International Conference for Learning Representations (ICML).

[22] Yu Y. Towards Sample Efficient Reinforcement Learning. Proceedings of the Twenty-Seventh International Joint Conference on Artificial Intelligence (IJCAI).

[23] Lehnert L., Laroche R., Seijen H.V. On Value Function Representation of Long Horizon Problems. Proceedings of the Thirty-Second AAAI Conference on Artificial Intelligence.

[24] Yao H., Zhang S., Zhang Y., Li J., Tian Q. (2016). Coarse-to-Fine Description for Fine-Grained Visual Categorization. IEEE Trans. Image Process..

[25] Ba J.L., Kiros J.R., Hinton G.E. (2016). Layer Normalization. arXiv.

[26] Blu T., Thevenaz P., Unser M. (2018). Linear Interpolation Revitalized. IEEE Trans. Image Process..

[27] Uchida S., Sakoe H. (2001). Piecewise Linear Two-Dimensional Warping. Syst. Comput. Jpn..

[28] Norouzi M., Bensio S., Chen Z., Jaitly N., Schuster M., Wu Y., Schuurmans D. Reward augmented maximum likelihood for neural structured prediction. Proceedings of the International Conference on Neural Information Processing Systems (NIPS).

[29] Kaufman D., Levi G., Hassner T., Wolf L. Temporal Tessellation: A Unified Approach for Video Analysis. Proceedings of the IEEE International Conference on Computer Vision (ICCV).

[30] Rochan M., Wang Y. Video Summarization by Learning from Unpaired Data. Proceedings of the IEEE Conference on Computer Vision and Pattern Recognition (CVPR).

[31] Apostolidis E., Metsai A.I., Adamantidou E., Mezaris V., Patras I. A Stepwise, Label-based Approach for Improving the Adversarial Training in Unsupervised Video Summarization. Proceedings of the 1st International Workshop on AI for Smart TV Content Production, Access and Delivery.

[32] Apostolidis E., Adamantidou E., Metsai A., Mezaris V., Patras I. Unsupervised Video Summarization via Attention-Driven Adversarial Learning. Proceedings of the International Conference on Multimedia Modeling (MMM).

